# Next-Generation Sequencing-Directed Therapy in Patients with Metastatic Breast Cancer in Routine Clinical Practice

**DOI:** 10.3390/cancers13184564

**Published:** 2021-09-11

**Authors:** Simona Bruzas, Sherko Kuemmel, Hakima Harrach, Elisabeth Breit, Beyhan Ataseven, Alexander Traut, Anna Rüland, Athina Kostara, Ouafaa Chiari, Christine Dittmer-Grabowski, Mattea Reinisch

**Affiliations:** 1 Interdisciplinary Breast Unit, Kliniken Essen-Mitte, 45136 Essen, Germany; S.Bruzas@kem-med.com (S.B.); S.Kuemmel@kem-med.com (S.K.); H.Harrach@kem-med.com (H.H.); E.Breit@kem-med.com (E.B.); A.Rueland@kem-med.com (A.R.); A.Kostara@kem-med.com (A.K.); O.Chiari@kem-med.com (O.C.); C.Dittmer-Grabowski@kem-med.com (C.D.-G.); 2 Department of Gynecology with Breast Center, Charité-Universitätsmedizin Berlin, Corporate Member of Freie Universität Berlin and Humboldt-Universität zu Berlin, 10117 Berlin, Germany; 3 Department of Gynecology and Gynecologic Oncology, Kliniken Essen-Mitte, 45136 Essen, Germany; B.Ataseven@kem-med.com (B.A.); A.Traut@kem-med.com (A.T.); 4 Department of Obstetrics and Gynecology, University Hospital, LMU Munich, 81377 Munich, Germany; 5 Brustzentrum, St. Marienhospital, 52353 Düren, Germany

**Keywords:** breast cancer, metastatic, next-generation sequencing, NGS, molecular profiling, targeted therapy, precision medicine

## Abstract

**Simple Summary:**

In earlier times, for patients with breast cancer who have metastases in distant organs, e.g., the lung, the mainstay of treatment was confined to chemotherapy. In recent years, additional therapeutic options have evolved as high-throughput commercial testing can identify alterations in genes, which are associated with cancer. Some of the identified genetic changes in each patient might be matched to a targeted therapy if the respective therapeutic agent is available. In this study, the implementation of this approach into routine clinical practice was investigated. All 95 patients with metastatic breast cancer had cancer-related genetic alterations, and, in 63 of them, these could be, in theory, matched to a genomically directed therapy. Out of 30 patients who were assigned to this therapy, 13 (43.3%) experienced an improved relapse-free period and 19 patients were still alive one year after molecular testing, in contrast to 15 out of 65 patients who received the standard therapy.

**Abstract:**

Next-generation sequencing (NGS) followed by matched therapy has opened up new therapeutic options to patients with metastatic breast cancer (mBC). Here we report our experience with this approach in everyday clinical practice. This retrospective study included 95 patients with mBC who were genotyped with the FoundationOne^®^ (CDx) assay in a commercial molecular pathology laboratory. Genetic alterations were identified in all tumor specimens, and 83 patients (87.4%) had a median of 2 (range, 1–6) potentially actionable alterations. A multidisciplinary tumor board recommended genomically guided therapy to 63 patients, 30 of whom received such treatment. Everolimus (*n* = 15) and anti-human epidermal growth factor receptor 2 (HER2) therapy (*n* = 6) were most frequently administered. The ratio of progression-free survival (PFS) under NGS-based therapy to PFS under the last line of standard therapy prior to NGS was >1.3 in 13 (43.3%) patients, indicative of a clinical benefit to NGS-directed therapy. One-year overall survival rates were 22.7% (95% CI, 6.5–44.4) in 65 patients allocated to the standard therapy versus 62.9% (95% CI, 41.6–78.2) in 30 patients receiving the matched therapy. In conclusion, NGS-matched treatment improved the clinical outcomes in a subgroup of mBC patients.

## 1. Introduction

The advent of commercially available, high-throughput genomic sequencing technologies has not only led to a tremendous increase in knowledge regarding the molecular profile of patients with breast cancer [1] but has also enabled the identification of potentially actionable targets [2]. This has been especially valuable for patients with metastatic breast cancer (mBC), for whom, in earlier times, therapeutic options were mainly limited to a few lines of chemotherapy [3], with rather poor outcomes. In recent years, new targeted therapeutics, e.g., cyclin-dependent kinase (CDK) 4/6 inhibitors, have created further options for patients who are either resistant to conventional therapy or unable to tolerate it [4,5]. In recently published clinical trials, several target-based agents, such as the immune checkpoint inhibitors atezolizumab [6] and pembrolizumab [7], have improved progression-free survival (PFS) in metastatic triple-negative breast cancer (TNBC). Likewise, the poly (ADP-ribose) polymerase (PARP) inhibitor olaparib is reserved for human epidermal growth factor receptor 2 (HER2)-negative mBC with germline mutations in *BRCA1* and *BRCA2* [8]. However, off-label therapy with olaparib in a patient with a *BRCA1/2* wild-type status but a germline *PALB2* mutation, as identified by next-generation sequencing (NGS), resulted in a remarkable response [9]. Even beyond the scope of case reports, the use of companion diagnostics covering a large set of cancer-related genes, followed by on-label or off-label genotype-matched treatment, has prolonged PFS in about 40% of patients with refractory disease [10,11], or increased the objective tumor response rate [12] in patients with a variety of advanced solid tumors. Some alterations detected by molecular profiling, e.g., deletion of *PIK3CA* exons 7, 9, and 20 with a therapeutic recommendation for alpelisib in patients with mBC, are listed in clinical guidelines [13].

Despite the promises held by molecular profiling in combination with precision cancer medicine, its implementation in everyday clinical practice has faced several challenges [14], including a lack of standardization regarding both the interpretation of complex genomic data [15,16] as well as software tools aiming to assist in the therapeutic interpretation [17]. Reimbursement issues [18] and problems with timely molecular testing in patients with progressive disease [19] have hampered successful clinical implementation as well. In addition, about 40% of all variants found by NGS are variants of uncertain significance (VUS) without a clear clinical indication [20].

The aim of this retrospective study was to evaluate NGS-based tumor profiling with the FoundationOne assay^®^ (and CDx) and the corresponding treatment decisions in patients with mBC, as well as to determine the clinical outcomes in patients who received the sequencing-matched or standard therapy in real-world settings.

## 2. Materials and Methods

### 2.1. Patients

This retrospective analysis included 95 patients with mBC who were treated at the Interdisciplinary Breast Unit of the Kliniken Essen-Mitte (KEM) from February 2018 to May 2020 and who were offered NGS. Molecular profiling was primarily considered when there was a lack or exhaustion of therapeutic recommendations according to the clinical practice guidelines.

### 2.2. Ethics Statement

Patients provided written informed consent for NGS and publication of their anonymized data. Retrospective studies conducted in Nordrhein-Westfalen (Germany) do not require formal ethics committee approval according to Section 15 of the respective medical association’s professional code of conduct.

### 2.3. Next-Generation Sequencing

Formalin-fixed, paraffin-embedded tumor-containing specimens were sent to the commercial molecular pathology laboratory Molekularpathologie Südbayern (Penzberg, Germany) for NGS. Details listed in the FoundationOne^®^ (CDx) reports, which were obtained by this laboratory, were used for this retrospective analysis. Biopsy tissue from distant tumor sites was preferably selected when feasible. Extracted DNA from tumor samples was subjected to NGS utilizing the hybrid capture-based FoundationOne^®^ or, from November 2018 onwards, FoundationOne^®^ CDx assay (Foundation Medicine Inc., Cambridge, MA, US), as previously described [21]. NGS was conducted for exons of 315 genes and introns of 28 genes (FoundationOne^®^) or for exons of 324 genes and introns of 36 genes (FoundationOne^®^ CDx), which are frequently altered in various solid tumors. The indicated genomic regions were investigated for base substitutions, insertions, deletions, copy number variants, rearrangements, microsatellite instability, and tumor mutational burden (TMB). NGS-based therapies listed in the FoundationOne^®^ (CDx) reports received prior to 10/2018 had approval in Germany while those listed in reports received after 11/2018 had approval in the European Union. A genetic alteration was regarded as potentially actionable when a matched therapeutic therapy was listed in the respective FoundationOne^®^ (CDx) report.

### 2.4. Multidisciplinary Tumor Board

At least six physicians with different medical specialties (senologist, gynecologic oncologist, radiologist, palliative care specialist, radiotherapist, and pathologist) attended the weekly tumor board (TB) meeting of the Interdisciplinary Breast Unit at the KEM. The role of the TB was to review clinical data, treatment history, performance status, NGS-based treatment recommendations, and genetic profile of each cancer patient and thereafter determine the best treatment strategy, including evaluation of the on-label and off-label therapies as well as participation in appropriate clinical studies. Off-label therapies were either therapies approved for other cancer types besides breast cancer or in a different tumor-receptor subtype than the one exhibited by the patient with mBC, or the patient had a mutation in a different gene than the one indicated for targeted treatment. Such off-label therapies usually required an application and approval for cost coverage by the respective health insurance company. In case the TB determined to proceed with NGS-based medication, patients received this selected targeted therapy at the time of disease progression, provided that the patient’s health status allowed a new line of treatment.

### 2.5. Response Assessment and Clinical Outcomes

Response to therapy was evaluated according to the RECIST 1.1 criteria [22]. PFS was defined as the time from initiation of either NGS-based or standard therapy to the date of either disease progression or death from any cause or last contact (censored), whichever occurred first. The PFS ratio (PFS2/PFS1) was calculated as PFS under NGS-guided therapy (PFS2) to PFS under conventional therapy (PFS1), directly preceding testing with NGS; a PFS ratio > 1.3 was regarded as an improvement in PFS under NGS-based therapy [23] and suggestive of a clinical benefit [10]. Overall survival (OS) was defined as the time from the first day of treatment after receipt of the test results of either sequencing-based or standard therapy to the date of death from any cause or last contact (censored). The receipt of the test results and subsequent therapy recommendations were considered a turning point in the treatment management of patients.

## 3. Results

### 3.1. Patient Characteristics

This retrospective analysis included 95 female patients with histologically confirmed mBC who were offered NGS to evaluate the potential targeted treatment options (Table 1). Eighty patients (84.2%) had secondary mBC and evolved distant metastases during the progression of their disease, whereas 15 patients (15.8%) presented with primary mBC.

The median age at initial diagnosis of breast cancer for the whole cohort was 49 years (range, 21–80); patients with primary mBC had a median age at diagnosis of 55 years (range, 29–80). The most prevalent tumor receptor subtype at initial breast cancer diagnosis was hormone receptor (HR) positive (HR+)/HER2 negative (HER2−) (56.8%). In 11 patients, the receptor status changed during the course of the disease and they exhibited triple-negative breast cancer (TNBC) at a later stage but not at the onset of the disease. All patients had received at least one line of therapy since the onset of recurrent disease prior to molecular profiling with NGS, and 18 patients had been assigned to five or more lines of therapy.

In all patients, one specimen was subjected to NGS with the FoundationOne^®^ (CDx) assay. Tumor-containing samples originated from several distant sites (Table 1) or the primary tumor in case it was not feasible to obtain an adequate specimen from the metastatic lesion.

### 3.2. Genetic Alterations Identified by Next-Generation Sequencing

A total of 1461 genetic alterations were detected in 95 patients, with a median of 14 (range, 3–47) alterations per patient (Figure S1) and a median of 14 (range, 2–29) alterations in 35 primary tumors. According to the original FoundationOne^®^ (CDx) reports, which were obtained by a commercial molecular pathology laboratory as part of routine treatment, 874 (59.8%) alterations were classified as variants of uncertain significance (VUS) (Table S1).

**Figure 1:**
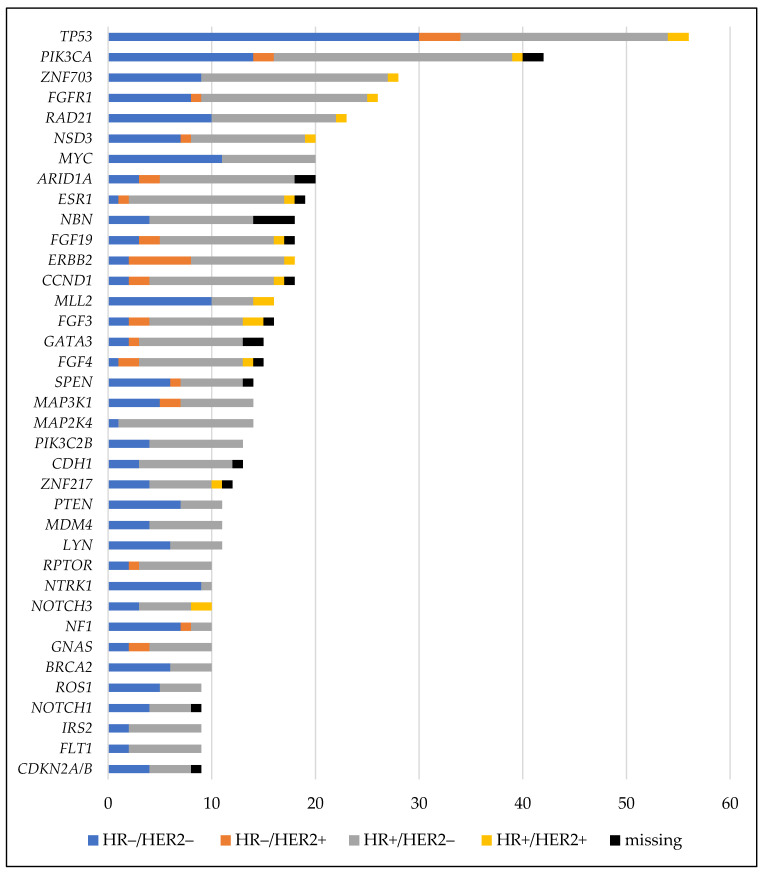
Genes that were altered ≥9 times in a cohort of 95 patients with metastatic breast cancer according to the tumor receptor subtype of the specimen subjected to genotyping. HER2, human epidermal growth factor receptor-2; HR, hormone receptor.

The most frequently altered genes in our cohort were *TP53* (56 alterations/55 patients), *PIK3CA* (42 alterations/35 patients), *ZNF703* (28 alterations/26 patients), *FGFR1* (26 alterations/24 patients), and *RAD21* (23 alterations/23 patients) (Table S1). HR-negative (HR−)/HER2− was the most prominent receptor subtype in the cohort with *TP53* mutations and HR+/HER2− in patients with alterations in *PIK3CA*, *ZNF703*, *FGFR1*, and *RAD21* (Figure 1).

### 3.3. Actionable Alterations Identified by Next-Generation Sequencing (NGS) and NGS-Based Therapy Options with Evaluation of On-Label and Off-Label Treatment

**Figure 2:**
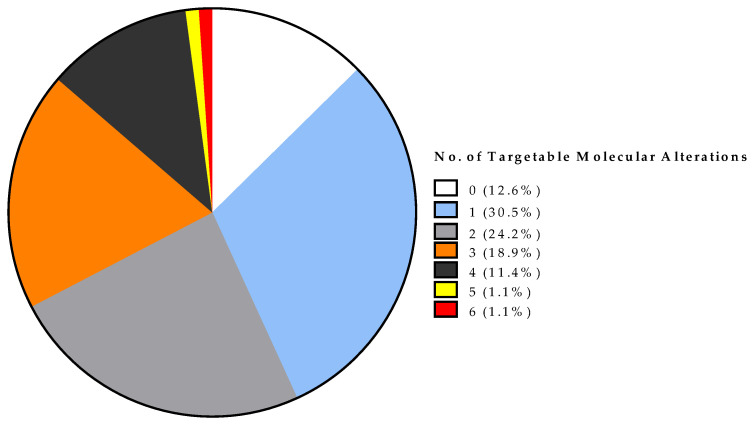
Targetable molecular alterations per patient in 95 women with metastatic breast cancer.

For 83 of 95 (87.4%) patients, 184 sequencing-matched therapies (median of 2; range, 1–6) were listed in the FoundationOne^®^ (or CDx) report either for breast cancer only (*n* = 29, 15.8%), for breast cancer and other cancer types (*n* = 95, 51.6%), or for other tumor types only (*n* = 60, 32.6%) (Table S1). Twelve (12.6%) patients did not present any actionable targets in their tumor specimen (Figure 2).

Of the 184 sequencing-matched therapies, 77 (41.8%) were on-label and 107 (58.2%) off-label therapies (Table S2). The mTOR inhibitor everolimus was indicated as a therapy for 62 actionable targets in 38 patients. Most actionable alterations were in *PIK3CA* (*n* = 38), with 21 of these in patients with HR+HER2− (Figure S2). Alpelisib as a targeted therapy was not yet approved for mBC during the conduct of the study for patients harboring a *PIK3CA* mutation. For 20 of the 22 patients with *FGFR1* or *FGFR2* amplification, off-label therapy with the multiple tyrosine kinase inhibitor pazopanib was indicated in the report. The CDK4/6 inhibitors ribociclib, palbociclib, and abemaciclib are approved for HR+/HER2− mBC and were allocated to all (*n* = 17) cases with *CCND1* amplification; however, four of these were either HR− or HER2+ and therefore CDK4/6 inhibitors were regarded as off-label therapy. Amplification or missense mutations in *ERBB2* were discovered in 13 instances and 11 patients, suggestive of on-label (histologically HER2+) or off-label (histologically HER2−) treatment with anti-HER2+ therapy (e.g., lapatinib, pertuzumab, trastuzumab emtansine) in all cases. Clinically relevant *ESR1* mutations were indicative of on-label therapy with fulvestrant in HR+/HER2− mBC.

### 3.4. Tumor Board Recommendations and Next-Generation Sequencing-Based Therapy

**Figure 3:**
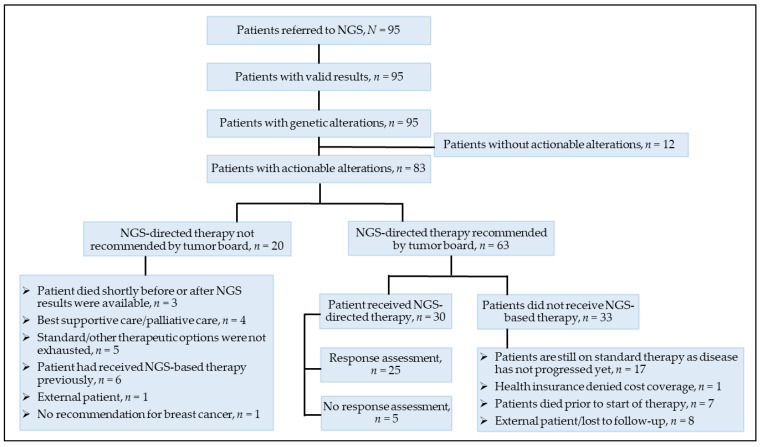
Patient flow of 95 patients who underwent molecular profiling with the FoundationOne^®^ (CDx) assay as part of their routine clinical practice. NGS, next-generation sequencing.

Discussion of the patient history, clinical parameters, and NGS-based therapy recommendations resulted in approval by the multidisciplinary TB of 36 on-label and 46 off-label agents of precision medicine in 63 (66.3%) patients (Table S2), implying application for cost coverage by the respective health insurance of 25 patients. In 14 patients, this was not done (yet), as they either died shortly after the results from the NGS were available (*n* = 2), were lost to follow-up (*n* = 4), or were continued on standard therapy as the disease has not progressed yet (*n* = 8). Out of 11 cases with cost applications, the health insurance reimbursed the costs for eight of these. Targeted therapy was most often recommended for molecular alterations in *PIK3CA* (*n* = 27), *ERBB2* (*n* = 8), *PTEN* (*n* = 8), *FGFR1* (*n* = 5), and *ESR1* (*n* = 5). The PARP inhibitor olaparib was recommended for four patients with missense mutations in *PALB2* and two patients with alterations in *BRCA1*. Despite having actionable mutations, 20 patients did not receive a recommendation for a matched therapy. The reasons for this were varied, as depicted in Figure 3.

**Figure 4:**
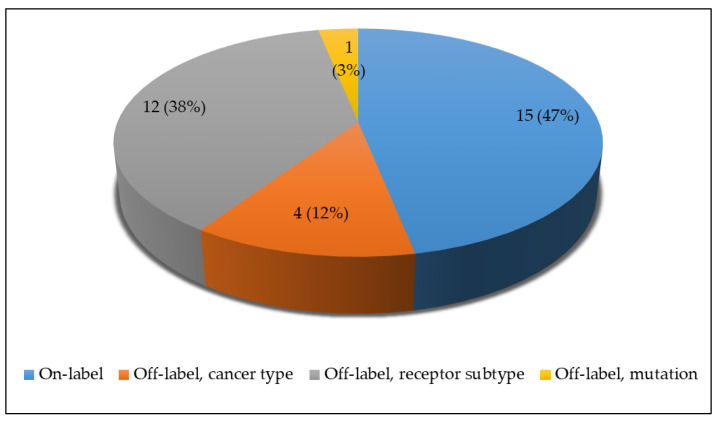
A total of 32 NGS-based therapies were recommended to 30 patients. Of the 17 off-label therapies, 12 were approved in a different tumor receptor subtype than the one exhibited by the patient, 4 were approved in different cancer types, and 1 was approved for a different mutation than the ones detected in the respective patient.

After a median of 9 days (range, 1–430) after the test results were delivered, 30 (31.6%) patients received NGS-directed therapy, and two patients (nos. 16 and 26) received two different lines of NGS-based therapy. Of the 32 agents, 15 were on-label and 17 were off-label (Figure 4). Patient no. 55, who inherited a genetic alteration in *PALB2*, was assigned to olaparib, which is approved for patients with a *BRCA1* or *BRCA2* germline mutation.

Six patients with de novo mBC (nos. 29, 43, 49, 58, 79, and 86) were assigned to NGS-based therapy after failure to one (*n* = 4) or two (*n* = 2) previous lines of systemic therapies, whereas patients with secondary mBC received matched therapy after a median of three (mean 3.5; range, 1–6) lines of standard therapy after the onset of recurrent malignancy (Table 2).

### 3.5. Clinical Outcomes

**Figure 5:**
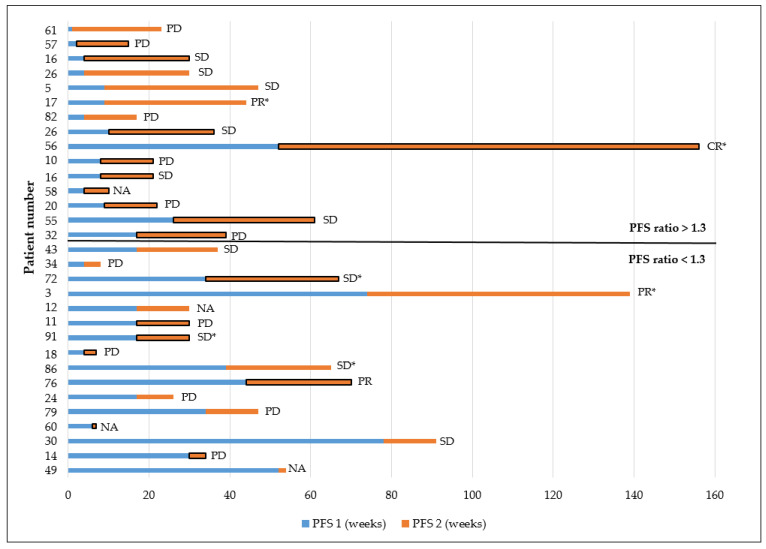
Progression-free survival (PFS) under the immediate last standard treatment line (PFS1, blue bar) before next-generation sequencing (NGS), followed by NGS-recommended treatment (PFS2, orange bar). Framed bars represent off-label therapy and * denotes ongoing treatment with targeted therapy. In four patients, the response assessment was not available (NA) as the patient died before the response could be evaluated. CR, complete response; PD, progressive disease; PR, partial response; SD, stable disease.

Fifteen out of 32 (46.9%) NGS-based therapies led to a PFS ratio > 1.3; 10 of these 15 (66.7%) were off-label agents (Figure 5).

Thirteen out of 30 (44.3%) patients allocated to NGS-based therapy had a PFS ratio > 1.3, suggestive of a clinical benefit. These included one patient each with CR or PR, four patients with SD, and six patients with PD, all of whom exhibited PFS of at least 13 weeks under NGS-based therapy. One patient with a PFS ratio of 1.5 died before a response could be evaluated. Another four patients with PR or SD and a current PFS ratio < 1.0 are still receiving NGS-based treatment. Irrespective of targeted therapy, complete loss of HR (*n* = 8) or progesterone receptor loss (*n* = 5; nos. 34, 61, 24, 12, and 29) during disease progression to distant disease seems to be an unfavorable event as eight patients had PD and another three had to discontinue therapy due to poor health status or death (Table 2).

One patient presented with a complete response (CR) and is still under treatment with the immune checkpoint inhibitor pembrolizumab (Table 2). This patient had three genetic alterations (amplification of *CD273*, *CD274*, and *PDCD1LG2*), indicative of off-label treatment with pembrolizumab. A partial response (PR) was obtained in three patients, stable disease (SD) in nine, and progressive disease (PD) in twelve patients. In another five patients, of which three had primary mBC, response assessment was not feasible as the treatment had to be discontinued due to the poor health status (*n* = 1) or death (*n* = 4) of the patient (Table 2). CDK4/6 inhibitors induced PR or SD in two patients with *CCND1* amplification; two other cases with *FGFR1* amplification exhibited SD during treatment with pazopanib. The best response to anti-HER2 therapy was PR in two and SD in three patients; two of the latter had a negative HER2−status based on immunohistochemistry. The best response to everolimus either as a single agent or in combination therapy was SD in three patients. Ten patients continued to advance to PD under therapy with everolimus; however, five of them had a PFS ratio > 1.3.

The median OS in the whole cohort was 40.3 weeks and was found to be significantly improved in patients who received NGS-matched therapy (*n* = 30; 69.1 weeks) in comparison to those who received the standard therapy (*n* = 65; 39.7 weeks; *p* = 0.039 by log-rank test) (Table 3).

The 1-year OS rate, after the test results were available, in these three cohorts was 44.0% (*n* = 95), 22.7% (*n* = 65), and 62.9% (*n* = 30), respectively. The clinical characteristics between these patient groups did not differ significantly, except for the tumor receptor subtype at initial diagnosis (Table S3).

## 4. Discussion

In this retrospective analysis, we evaluated the effectiveness of NGS-based treatment recommendations in real-world routine management of patients with mBC. In all patients, at least three genomic alterations were identified by NGS with the FoundationOne^®^ (CDx) assay, targeting 324 genes. In 83 of 95 (87.4%) patients, a median of two (range, 1–6) potentially actionable targets were indicated, whereas in 12 patients (12.6%) no actionable target was found. Thirty patients received NGS-matched therapy and in 13 of them (43.3%), a PFS ratio > 1.3 was suggestive of a clinical benefit.

In a prospective study including 404 patients with mBC, molecular profiling was performed with three assays covering panels with a substantially lower number of cancer-related genes [25]. Only in 46% of patients at least one alteration was found, emphasizing the need for comprehensive genotyping. The median number of genomic alterations, including VUS, in the tumors of the patients included in our analysis was 14 (range, 3–47). The most frequently altered genes identified in our cohort were *TP53* (57.9%), *PIK3CA* (36.8%), *ZNF703* (27.4%), and *FGFR1* (25.3%). In comparison, NGS with gene panels comprising more than 300 cancer-associated genes in a study cohort encompassing 1324 patients with mBC identified alterations in *TP53* in 41.5%, *PIK3CA* mutations in 35.4%, and *FGFR1* alterations in 13.2% [26]. In another investigation of mBC, a high prevalence of *ZNF703* amplifications was identified after genotyping [27], suggesting genomic similarities in key drivers between our cohort and other populations with mBC. In more than 80% of the specimens from our analysis, NGS revealed potentially actionable alterations, most commonly located in *PIK3CA*, *FGFR1*, *CCND1*, *ERBB2*, *ESR1*, and *PTEN*.

The multidisciplinary TB recommended NGS-matched therapy in 63 patients of whom 30 were started on a total of 32 agents. In total, 17 were off-label treatment and 10 (58.8%) of these induced a PFS ratio > 1.3; 15 agents were on-label treatment and 5 of these (33.3%) induced a PFS ratio > 1.3, demonstrating a clinical benefit of on-label and off-label therapy determined by NGS. Out of 30 patients, 13 (43.3%) demonstrated a PFS ratio > 1.3; other investigations with a similar intention showed comparable results (44% [28] and 44.4% [11]) or a lower proportion (30%, [29]) of mBC patients with improved PFS under matched therapy. In the 15 cases who started therapy with the mTOR inhibitor everolimus, mostly in combination with exemestan, the best response was SD in 3 patients. Of note, these patients had concurrent amplification of *FGFR*1 or *FGFR2* and alterations in the genes allocated to the PI3K/AKT/mTOR pathway. It has been recently described that mBC patients with this genetic makeup had a better response to agents targeting the PI3K/AKT/mTOR pathway [30] and it might be therefore worthwhile taking this aspect into consideration regarding treatment decisions. The other eleven patients with response assessment available had PD; however, five of these with PFS ratio > 1.3 still experienced a clinical benefit. In three of these five patients, NGS therapy was based on the *PIK3CA* hotspot mutation (H1047R) belonging to ESMO Scale for Clinical Actionability of molecular Targets (ESCAT) level IA alterations, for which the respective targeted treatment has been evaluated in clinical trials [24]. Median OS in the patient group who received NGS-directed therapy was significantly better than in the 65 patients who did not receive matched therapy (69.1 weeks versus 39.7 weeks, *p* = 0.039). As these results, however, were based on a retrospective analysis of two patient groups that were not matched according to their baseline characteristics and disease course, they should be regarded with caution.

It has been previously pointed out that only in a subgroup of advanced cancer patients who initially had been assigned to molecular profiling, sequencing-directed therapy was pursued [11,31]. In our analysis, 31.6% of the total cohort and 36.1% of those with actionable alterations were started on matched therapy. While a lack of reimbursement for [18] or access to sequencing-directed therapy [32] were some of the barriers preventing treatment with matched therapy in other evaluations, this was the case only for one patient in our evaluation. On the other hand, the delivery of NGS-based agents to patients included in our analysis was hampered for various reasons. A total of 22 patients (23.2%) were still on standard therapy, and for 17 patients the TB-recommended NGS-based therapy if the disease progresses, suggesting future referral to matched targeted therapy. Other patients had experienced PD during NGS-based therapy earlier on in their disease history. Inherent to an investigation of patients with late-stage disease is that several patients with a low performance status had to be allocated to supportive care or died shortly after NGS was done, or prior to or shortly after initiation of matched therapy. This was also a major issue elsewhere [31] and emphasizes the need for timely initiation of genomic sequencing in patients with mBC, preferentially in those with an acceptable performance status.

A potential drawback of applying modern sequencing technologies to patients with mBC is that formalin-fixed, paraffin-embedded specimens from distant tumor sites might not be available with sufficient quality to yield DNA fulfilling the minimum requirements for sequencing technologies, and the primary tumor is subsequently investigated instead. However, the primary tumor of initially untreated breast cancer patients might not always reflect the later genomic evolution, leading to distant spread [33]. In this retrospective study of 35 cases in whom the DNA of the primary breast tumor was subjected to molecular analysis, 27 originated from archival tumor specimens of patients who were initially metastasis-free. This presents a limitation of our investigation but at the same time reflects the real-world experience. Alternatively, liquid biopsies could be used for NGS in order to ensure easy access to a tumor-containing sample representative of the metastatic disease [34], but this approach is, however, currently not covered by German health insurances and therefore not routinely applied yet.

## 5. Conclusions

About one-third of patients with mBC who underwent molecular profiling received NGS-based therapy and presented with improved clinical outcomes compared to patients who did not receive therapy matched to genomic alterations. More than 40% of the patients who received NGS-therapy had a clinical benefit. Major hurdles to successful implementation of genotyping into the metastatic setting is that patients had previously failed on NGS-based therapy or were not able to undergo further therapy because of poor performance status or death, emphasizing the need for timely initiation of molecular testing.

## Figures and Tables

**Table 1:** Clinical and demographic characteristics of 95 patients with metastatic breast cancer.

Variable		No.	%
Patients		95	100
Age (years) at initial diagnosis of BC	median (range)	49 (21–80)	
Age (years) when NGS was performed	median (range)	55 (25–82)	
Tumor receptor subtype at diagnosis	HR+/HER2−	54	56.8
	HR−/HER2−	27	28.4
	HR+/HER2+	7	7.4
	HR−/HER2+	4	4.2
	missing	3	3.2
Tumor receptor subtype of recurrent tumor site	HR+/HER2−	48	50.5
	HR−/HER2−	38	40.0
	HR+/HER2+	2	2.1
	HR−/HER2+	5	5.3
	missing	2	2.1
Number of systemic therapy lines for recurrent disease prior NGS testing	1	27	28.4
	2	25	26.3
	3	13	13.7
	4	12	12.6
	≥5	18	18.9
Biopsy site for NGS	primary tumor	35	36.8
	lymph node	19	20.0
	skin	9	9.5
	liver	17	17.9
	lung/pleura	5	5.3
	other distant sites	10	10.5

BC, breast cancer; CI, confidence interval; HER2, human epidermal growth factor receptor-2; HR, hormone receptor; NGS, next-generation sequencing.

**Table 2:** Next-generation sequencing (NGS)-based treatment and indication of best response in 30 patients with mBC. Underlined gene alterations represent the ESMO Scale for Clinical Actionability of molecular Targets (ESCAT) level IA [24]. The progression-free survival (PFS) ratio was calculated as PFS under the NGS-recommended treatment (PFS2) to PFS under the immediate previous standard treatment line (PFS1).

Patient No.^1^	Gene	Alteration	Receptor Status of NGS Sample	Previous Lines (No.) of Therapy (M1)	NGS-Based Therapy	Best Response	PFS Ratio
61	*PIK3CA*	H1047R	HR+HER2−	3	Everolimus + exemestan	PD	22.00
57	*ERBB2*	S310F	HR+HER2−	2	Docetaxel + trastuzumab + pertuzumab	PD	6.50
16 ^2^	*AKT1*	E17K	HR−HER2− ^3^	1	1. Everolimus + exemestan	SD	6.50
	*FGFR1*	amplification			2. Pazopanib	SD	1.63
26 ^2^	*PTEN*	loss	HR+HER2+	6	1. Everolimus + fulvestrant	SD	6.50
	*FGFR1*	amplification			2. Pazopanib	SD	2.60
5	*ERBB2*	D769Y	HR−HER2+ ^3^	6	Trastuzumab emtansine	SD	4.22
17	*ERBB2*	amplification	HR−HER2+	2	Trastuzumab deruxtecan	PR	3.89
82	*PIK3CA*	H1047R	HR+HER2−	4	Everolimus + exemestan	PD	3.25
56	*CD274* (*PD-L1*), *CD273*, *PDCD1LG2*	amplification	HR−HER2+	3	Pembrolizumab + nab-paclitaxel, after 1 year only pembrolizumab	CR	2.00
10	*AKT3*	amplification	HR−HER2−	2	Everolimus	PD	1.63
58	*CCND1*	amplification	HR−HER2−	1	Palbociclib + letrozol	Patient died before response could be evaluated.	1.50
20	*PIK3CA*	Q546R	HR−HER2− ^3^	3	Everolimus + exemestan	PD	1.44
55	*PALB2*	E830fs * 21	HR+HER2− ^4^	3	Olaparib	SD	1.35
32	*PIK3CA*	H1047R	HR−HER2− ^3^	2	Everolimus + exemestan	PD	1.35
43	*ERBB2*	amplification	HR−HER2+	1	Trastuzumab emtansine	SD	1.18
34	*PIK3CA*	C420R	HR+HER2−	4	Everolimus + exemestan	PD	1.00
72	*ERBB2*	T652A	HR+HER2−	1	Lapatinib + capecitabine	SD	0.97
3	*CCND1*	amplification	HR+HER2−	2	Palbociclib + fulvestrant	PR	0.88
12	*ESR1*	V422del	HR+HER2−	2	Fulvestrant	Patient died before response could be evaluated.	0.76
11	*PIK3CA* *PTEN*	N345Kloss	HR−HER2+ ^3^	3	Everolimus	PD	0.76
91	*PIK3CA*	N345K	HR−HER2+	5	Everolimus + exemestan	SD	0.76
18	*PIK3CA*	H1047L	HR−HER2− ^3^	4	Everolimus + exemestan	PD	0.75
86	*ESR1*	D538G	HR+HER2−	1	Fulvestrant	SD	0.67
76	*ERBB2*	G776>VC	HR+HER2−	3	Docetaxel + trastuzumab + pertuzumab	PR	0.59
24	*ESR1*	Y537C	HR+HER2−	4	Fulvestrant	PD	0.53
79	*PIK3CA*	L113del	HR+HER2−	1	Everolimus + exemestan	PD	0.38
60	*FGFR1*	amplification	HR−HER2− ^3^	6	Pazopanib	Patient died before response could be evaluated.	0.17
30	*CCND1*	amplification	HR+HER2−	4	CDK4/6 inhibitor + exemestan	SD	0.17
14	*AKT1*	E17K	HR−HER2− ^3^	5	Everolimus + exemestan	PD	0.13
49	*PTEN*	splice site 165-2A>G	HR+HER2−	2	Everolimus + exemestan	Patient died before response could be evaluated.	0.04
29	*STK11*	G394_ * 434>?	HR+HER2−	2	Everolimus + exemestan	Therapy was discontinued due to poor health status of patient.	

^1^ The patient number originates from Table S1. ^2^ In between the two lines of NGS-based therapy the patients received a line of standard therapy. ^3^ The primary tumor had HR+HER2− or ^4^ HR+HER2+ receptor status at diagnosis (M0). CR, complete response; HER2, human epidermal growth factor receptor-2; HR, hormone receptor; PD, progressive disease; PR, partial response; SD, stable disease; *, stop codon.

**Table 3:** NGS-based treatment and follow-up.

Variable		No.	%
Time from receipt of test results to start of NGS-directed therapy (days) (*n* = 30)	median (range)	9 (1–430)	
Best response to NGS-directed therapy in patients (*n* = 30)	complete response	1	3.3
	partial response	3	10.0
	stable disease	9	30.0
	progressive disease	12	40.0
	not available	5	16.7
OS after initial diagnosis (months) (*n* = 95)	median (95% CI)	166 (80–251)	
Death of patients	yes	36	37.9
	no	59	62.1
OS after test results (weeks) (*n* = 95)	median (95% CI)	40.3 (19.8–60.7)	
	1-year OS rate (95% CI)	44.0 (29.6–57.6)	
OS after test results in patients receiving NGS-directed therapy (weeks) (*n* = 30)	median (95% CI)	69.1 (17.9–120.4)	
	1-year OS rate (95% CI)	62.9 (41.6–78.2)	
OS after test results in patients not receiving NGS-directed therapy (weeks) (*n* = 65)	median (95% CI)	39.7 (12.7–66.7)	
	1-year OS rate (95% CI)	22.7 (6.5–44.4)	

CI, confidence interval; NGS, next-generation sequencing; OS, overall survival.

## Data Availability

The data are contained within the article or Supplementary Material. Data presented in this study are available in Tables S1 and S2.

## Supplementary Materials

The following are available online at https://www.mdpi.com/article/10.3390/cancers13184564/s1, Figure S1: Number of genetic aberrations per patient identified by the FoundationOne® (CDx) assay, Figure S2: Genes with targetable molecular alterations according to tumor receptor subtype in 83 patients with metastatic breast cancer, Table S1: Genetic alterations identified by next-generation sequencing in 95 patients with metastatic breast cancer, Table S2: Actionable alterations and approval (+) of NGS-based therapy by tumor board (TB), Table S3: Clinical characteristics of 30 patients who received NGS-directed therapy versus 65 patients who did not receive matched therapy.
