# Supplementary material for: Next-Generation Sequencing-Directed Therapy in Patients with Metastatic Breast Cancer in Routine Clinical Practice
Source: Cancers (Basel). 2021 Sep 11;13(18):4564. doi: 10.3390/cancers13184564 (PMC8468801; doi:10.3390/cancers13184564)
Supplement: Supplementary file 1 [file cancers-13-04564-s001.zip › cancers-1336427-supplementary/Supplementary material/Supplementary Figures S1 + S2 and Tables S2 + S3.pdf]

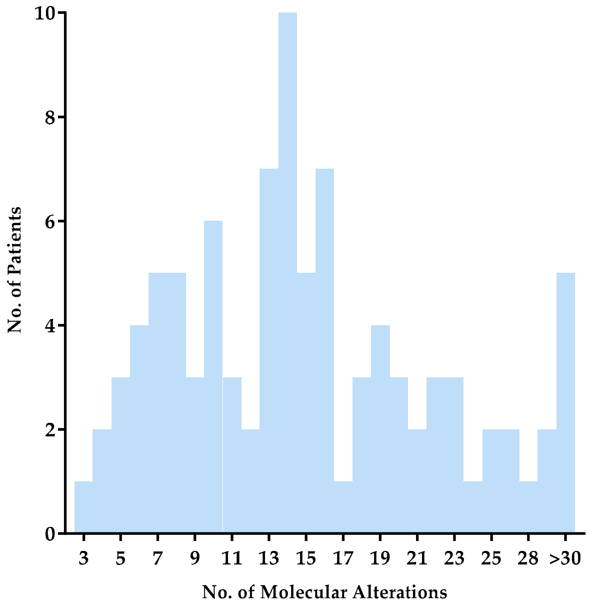

Figure S1. Number of genetic aberrations per patient identified by the FoundationOne® (CDx) assay.

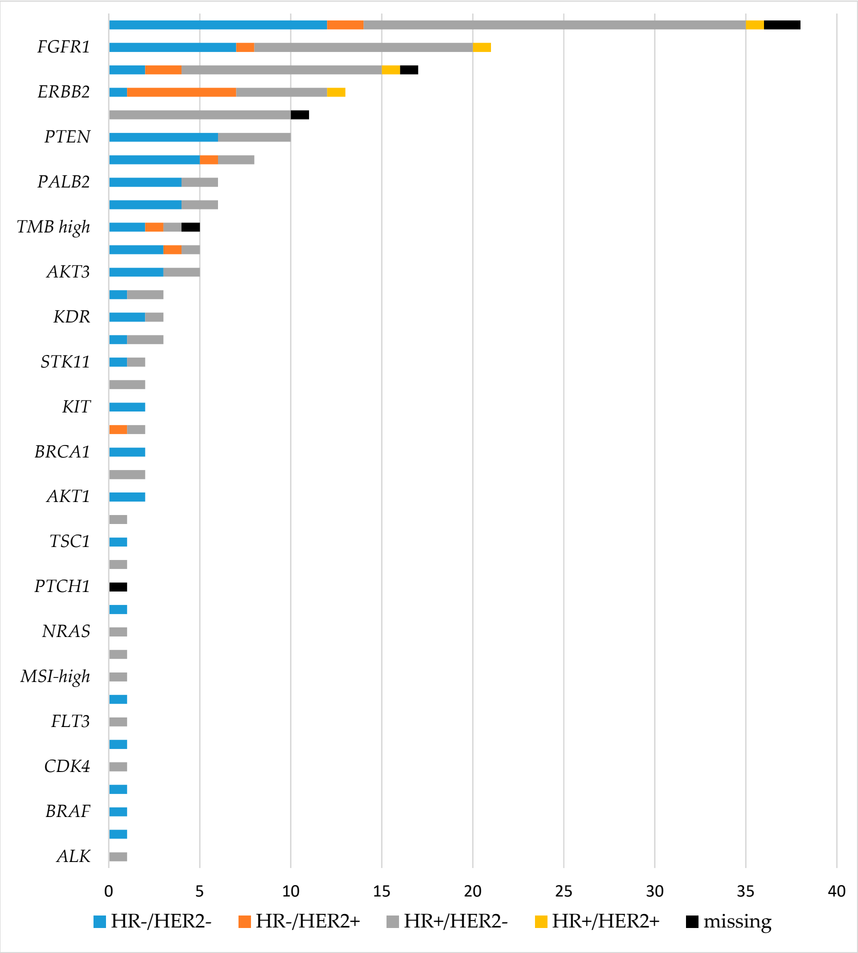

Figure S2. Genes with targetable molecular alterations according to tumor receptor subtype in 83 patients with metastatic breast cancer. *PIK3CA*, *FGFR1*, *CCND1*, *ERBB2*, and *ESR1* had >10 targetable alterations, mostly allocated to HR+/HER2- subtype except alterations in *ERBB2* which were most often present in patients with HR-/HER2+ disease. HER2, human epidermal growth factor receptor-2; HR, hormone receptor.

Deleted: ¶

Deleted: ¶

Table S2. Actionable alterations and approval (+) of NGS-based therapy by tumor board.

Genes with Actionable Alterations	Total	NGS-Recommended Therapies				TB-Approved Therapies
		Off-Label		On-Label		
		Total	TB+	Total	TB+	
AKT1	2	0	0	2	2	2
AKT3	5	2	2	3	1	3
ALK	1	1	0	0	0	0
ARAF	1	1	0	0	0	0
ATM	2	2	0	0	0	0
BRAF	1	1	1	0	0	1
BRCA1	2	1	1	1	1	2
BRCA2	6	4	0	2	0	0
CCND1	17	4	0	13	4	4
CD273	1	1	0	0	0	0
CD274	3	1	1	2	2	3
CDK4	1	0	0	1	0	0
EGFR	5	4	0	1	0	0
ERBB2	13	7	4	6	4	8
ESR1	11	0	0	11	5	5
FBXW7	2	1	0	1	1	1
FGFR1	21	21	5	0	0	5
FGFR2	1	1	0	0	0	0
FLT3	1	1	0	0	0	0
FLT4	1	1	0	0	0	0
KDR	3	3	0	0	0	0
KIT	2	2	0	0	0	0
KRAS	2	2	0	0	0	0
MSI-high	1	1	1	0	0	1
MTOR	1	0	0	1	0	0
NF1	8	8	0	0	0	0
NRAS	1	1	0	0	0	0
PALB2	6	6	4	0	0	4
PDCD1LG2	3	1	1	2	2	3
PDGFRA	1	1	0	0	0	0
PIK3CA	38	12	8	26	19	27
PTEN	10	7	5	3	3	8
PTCH1	1	1	0	0	0	0
RAD51c	1	1	1	0	0	1
STK11	2	1	0	1	1	1
TMB high	5	5	2	0	0	2
TSC1	1	1	0	0	0	0
TSC2	1	0	0	1	1	1
Total	184	107	36	77	46	82
Patients[1]	83	66	32	56	34	63

[1]Some patients had more than one actionable alteration in the same gene or actionable mutations in several genes. MSI, microsatellite instability; NGS, next-generation sequencing; TMB, tumor mutational burden; TB, tumor board.

Table S3. Clinical characteristics of 30 patients who received NGS-directed therapy versus 65 patients who did not receive matched therapy.

Variable		No.	%	No.	%	No.	%	P value
Patients		95	100	30	100	65	100	
Age (years) at diagnosis	median (range)	49 (21–80)		51 (21–80)		49 (24–74)		0.209
Age (years) when NGS was performed	median (range)	55 (25–82)		56 (25–82)		55 (26–81)		0.457
Metastatic status at diagnosis	M0	80	84.2	24	80.0	56	86.2	0.445
	M1	15	15.8	6	20.0	9	13.8	
Progression-free survival (months) in M0 patients after initial diagnosis	median (95% CI)	35.3 (28.0–43.0)		36.0 (21.6–50.4)		31.0 (22.0–40.0)		0.302
Tumor receptor subtype at diagnosis	HR+/HER2-	54	56.8	20	66.7	34	52.3	0.011
	HR-/HER2-	27	28.4	3	10.0	24	36.9	
	HR+/HER2+	7	7.4	4	13.6	3	4.6	
	HR-/HER2+	4	4.2	3	10.0	1	1.5	
	missing	3	3.2	0	0	3	4.6	
Tumor receptor subtype of recurrent tumor site	HR+/HER2-	48	50.5	16	53.3	32	49.2	0.098
	HR-/HER2-	38	40.0	10	33.3	28	43.1	
	HR+/HER2+	2	2.1	0	0	2	3.1	
	HR-/HER2+	5	5.3	4	13.3	1	1.5	
	missing	2	2.1	0	0	2	3.1	
Number of prior systemic therapy lines for recurrent disease	1	27	28.4	6	20.0	21	32.3	0.548
	2	25	26.3	8	26.7	17	26.2	
	3	13	13.7	6	20.0	7	10.8	
	4	12	12.6	5	16.7	7	10.8	
	≥ 5	18	18.9	5	16.7	13	20.0	
Biopsy site for NGS	primary tumor	35	36.8	10	33.3	25	38.5	0.944
	lymph node	19	20.0	6	20.0	13	20.0	
	skin	9	9.5	3	10.0	6	9.2	
	liver	17	17.9	7	23.3	10	15.4	
	lung/pleura	5	5.3	1	3.3	4	6.2	
	others	10	10.5	3	10.0	7	10.8	

CI, confidence interval; HER2, human epidermal growth factor receptor-2; HR, hormone receptor; NGS, next-generation sequencing.
